# Evaluating community engagement strategies in COVID-19: insights from a National Quasi-Experimental Intervention

**DOI:** 10.1186/s12889-025-24403-7

**Published:** 2025-08-26

**Authors:** Hamid Soori, Martha Orendu Oche Attah

**Affiliations:** 1 Faculty of Medicine, Cyprus International University, via Mersin 10, Nicosia, Cyprus; 2 College of Medicine, Dhofar University, Salaleh, Sultanate of Oman; 3 Department of Human Anatomy, Faculty of Basic Medical Sciences, University of Maiduguri, Maiduguri, Borno State Nigeria; 4 Department of Human Anatomy, Faculty of Medicine, Cyprus International University, via Mersin 10, Nicosia, Cyprus

**Keywords:** Epidemic, COVID-19, Control, Intervention, Iran

## Abstract

**Background:**

Community engagement is essential during crises such as the COVID-19 pandemic. However, the effectiveness of community engagement during this pandemic, especially in low- and middle-income countries, has not been seriously investigated.

**Objective:**

The effectiveness of a comprehensive intervention program in managing COVID-19 in Iran emphasizes community involvement and multifaceted strategies.

**Participants:**

All individuals who were admitted to hospitals and outpatient clinics across the country with suspected COVID-19 symptoms.

**Methods:**

A quasi-experimental study was conducted on the implementation of interventions (supportive, caring, and supervisory) by neighborhood-based teams during the COVID-19 epidemic in Iran. The evaluation took place four months later. Data from various inpatient and mortality sources was used, along with statistical-epidemiological analyses such as logistic regression analysis, and odds ratio.

**Results:**

Deaths per day declined from 479 to 75 within the study period. R_0_ decreased from 1.26 to 0.86. PCR tests reached from 661 to 1601 /100,000. The incidence rate of the disease reached 0.2 per hundred thousand people to 0.05 per hundred thousand people. The number of hospitalizations from COVID-19 decreased from 3044 to 417 before and after the community-based interventions.

**Conclusion:**

Epidemic management when combined with community participation can be very effective in crisis situations. Strengthening the disease care system and more supervision in the implementation of the strategy and having an effective relationship with the doctors of the private sector to comply with the national protocol, an effective step will be taken towards the control of this disease and finally its elimination.

## Introduction

In the last twenty years, the world has faced several major epidemics, such as SARS in 2003, Avian Influenza in 2005, Swine Flu in 2009, MERS in 2012, Ebola in 2014, and COVID-19 in 2020. These pandemics have greatly affected global health, economies, and societies, highlighting the need for strong preparedness and response strategies for infectious diseases [1]. Notably, the impacts of COVID-19 have been particularly challenging for low and middle-income countries, including Iran [2]. Understanding COVID-19 dynamics and intervention effectiveness is vital for improving public health responses.

In a short time, the novel coronavirus (SARS-CoV-2) has caused outbreaks in nearly all provinces of China and over 200 countries worldwide, including some in the Eastern Mediterranean region. The World Health Organization (WHO) declared it a global public health emergency. By early November 2024, there have been about 777 million cases and more than 7 million deaths [3].

The emergence of the novel coronavirus (SARS-CoV-2) escalated this challenge, causing outbreaks in nearly all provinces of China and over 200 countries worldwide, including several in the Eastern Mediterranean region. This rapid spread prompted the World Health Organization (WHO) to declare it a global public health emergency. By early November 2024, COVID-19 had resulted in approximately 777 million cases and more than 7 million deaths [3], significantly disrupting daily lives and economic conditions in affected communities [4].

Understanding the clinical indications of COVID-19—such as fever, cough, and dyspnea—is essential for accurate diagnosis and effective management. These symptoms, along with other manifestations like neurological and dermatological symptoms, underline the complexity of the disease [5, 6]. This knowledge is critical as it informs both clinical practice and public health responses.

To combat the spread of COVID-19, traditional public health measures—including quarantine, social distancing, hand hygiene, and mask usage—have proven vital. Additionally, Rapid Reaction Teams (RRTs), composed of interdisciplinary health personnel, play a crucial role in swiftly responding to outbreaks. Effective vaccines against SARS-CoV-2 are also essential, showing efficacy against confirmed infections and severe cases. However, disparities in vaccination coverage, particularly among children, necessitate a combined approach of therapeutic interventions and preventative efforts for optimal outcomes [7].

As COVID-19 spread in Iran, the country swiftly transitioned to community transmission. In response, the Ministry of Health established the National Corona Crisis Management Headquarters to issue guidelines aimed at controlling the pandemic. Strategies included isolating infected individuals, quarantining suspected cases, enforcing social distancing, and suspending certain healthcare services. The formation of a National Committee for Rapid Reaction on February 7, 2020, aimed to coordinate these efforts. Despite these initiatives, public adherence to preventive measures weakened over time, particularly with the arrival of the Delta variant in June 2021, which sparked outbreaks in southern provinces and initiated a fourth wave in southeast Iran [8].

A review of non-pharmaceutical interventions in Southeast Asia for controlling epidemics highlights measures like hand hygiene, mask-wearing, social distancing, lockdown, movement restrictions, amongst other preventative measures taken [9]. While these strategies are effective, their impact is limited when implemented in isolation, particularly in low and middle-income settings. Therefore, mass testing and isolating confirmed cases are crucial, as asymptomatic individuals can facilitate the virus’s spread. Integrating biomedical and social approaches is essential for more effective disease control, with community engagement playing a vital role. Results will guide health strategies to meet community needs and manage diseases in vulnerable groups. The goal is to improve COVID-19 control in Iran through community engagement, collaboration, and health equity.

Public health initiatives at the community level significantly influence outbreak control, emphasizing the need for targeted efforts. Addressing social determinants and inequalities is particularly crucial in disadvantaged neighborhoods [10]. Ultimately, collaboration and community involvement are essential for enhancing health equity and effectively responding to epidemics. The research problem examines the effectiveness of intervention programs in managing the COVID-19 epidemic in Iran, focusing on community involvement and collaboration.

The study evaluates a comprehensive intervention program’s effectiveness in controlling the COVID-19 epidemic, emphasizing community engagement, intersectoral collaboration, and a multifaceted approach to address various outbreak aspects in Iran.

## Methods

### Time frame of the intervention

This quasi-experimental study utilized a before-and-after design to assess the effectiveness of a multifaceted intervention program in controlling the COVID-19 epidemic in Iran during the 2021 outbreak. The intervention, implemented over a three-week period from July 3 to July 24, 2021, involved a rapid response team employing community engagement and intersectoral cooperation strategies.

### Components of the intervention strategy

The intervention strategy comprised three categories: supportive, caring, and supervisory. Volunteer forces were organized into neighborhood-based teams to implement these components during the COVID-19 epidemic, which began in November 2020. The study took place in Sistan-Balouchestan province, which includes seven main cities and has a total population of about 2.8 million. Key elements included public health processes to assess health and disease indicators, active case finding through contact tracing and education, and environmental health supervision to ensure compliance with safety standards. Additionally, case management involved mass testing, laboratory management, quarantine, isolation, and vaccination centers.

Three categories of interventions—supportive, caring, and supervisory—were implemented using volunteer forces organized into neighborhood-based teams during the COVID-19 epidemic in Iran, which began in November 2020. The intervention program comprised several key components, including public health processes that assess health and disease indicators in different zones; active case finding that involves contact tracing, education, and social mobilization to raise awareness, promote prevention, and distribute personal protective equipment; environmental and occupational health supervision to ensure compliance with safety standards; and case management, which includes mass testing, laboratory management, quarantine, isolation, and vaccination centers to manage cases effectively.

Optimizing health-care delivery involved assessing outpatient clinics, inpatient units, and ICU in both public and private sectors. This included managing treatment, ensuring oxygen supply, overseeing medication distribution, expanding capacity, addressing staffing needs, providing education, and offering family support. The intervention in social processes addressed the outbreak’s social dimensions by supporting low-income families, disseminating accurate information, managing border control, coordinating with medical universities, encouraging social participation, seeking charitable assistance, engaging local influencers, and reviving community mobilization to identify high-risk individuals.

The study tools were developed in accordance with the standards set by the national ministry of health. Public health experts confirmed the tool’s content and construct validity.

Trans-sectoral processes entailed collaboration with COVID-19 management officials at various administrative levels, temporarily closing public centers to reduce transmission, and managing traffic restrictions. Human resource mobilization aimed to maximize participation from different sectors, while logistical processes addressed the documentation and monitoring aspects of the intervention.

Logistic and documentation processes focused on the logistical and documentation aspects of the intervention. This involved leveraging existing technologies for monitoring and controlling the outbreak, establishing a daily registry and reporting system, ensuring adequate staffing, and protecting the health of team members. Thorough documentation of interventions and reporting at various levels were conducted to evaluate and refine the response strategy.

### Categorization of patients

Executive committees created operational plans for handling admissions during an outbreak, categorizing patients as confirmed or suspected cases per Ministry of Health guidelines. Confirmed cases had positive test results, while suspected cases presented with severe respiratory symptoms. Patients with atypical symptoms were also considered suspected cases. Those who refused testing or hospitalization for cultural reasons were isolated at home.

### Ethical considerations

This work was approved by the Research Ethics Committees of Shahid Beheshti University of Medical Sciences (Ethical and trial code: IR.SBMU.RETECH.REC.1401.013). Informed consent was obtained from all participants.

### Statistical analysis

Data collected over four months post-intervention were analyzed using descriptive statistics in Microsoft Excel and GraphPad prism (version 8). Mean differences were reported to reflect changes. The study outcomes included the total number of confirmed positive cases, the number of hospital discharges, and deaths among confirmed cases. Ratios calculated included the ratio of positive tests to total tests performed, vaccination coverage percentage, and death rate. Logistic regression analysis was conducted to assess the relationship between positive test results and various predictors. Data from various inpatient and mortality sources was used, along with statistical-epidemiological analyses such as logistic regression analysis, and odds ratio.

## Results

During the study period, a total of 2,514,911 individuals were admitted to hospitals and outpatient clinics with COVID-19 related symptoms. Out of these, 2,125,389 individuals (accounting for 84.5%), were ultimately discharged. Among those discharged, 796,372 were confirmed positive cases (representing 37.5% of the discharges), 494,746 were classified as suspected cases (making up 23.3%) and 834,271 tested negative (constituting 39.2% of the discharges). Additionally, it was noted that 58,441 cases (or 2.3%), required hospital admission during this period.

A total of 310,741 tests were conducted, translating to 111,978 tests per million people, with an overall positive result proportion of 28%. The reproduction number (R0) decreased from 1.26 to 0.86. The number of PCR tests increased from 661 to 1,601 per 100,000. The comparative analysis showed a notable change in the positivity ratio, decreasing from 36% pre-intervention to 21.7% post-intervention, resulting in a reduction of 14.3% (see Table 1). It is important to consider that individuals could be admitted and tested multiple times throughout the study. The overall percentage of positive results decreased after the intervention, indicating that there was a significant reduction in the positivity rate of tests conducted, particularly in PCR tests.

**Table 1:** Ratio of positive test results to total tests performed before and after the intervention

	Before				After				Difference
	Num. of tests	Num. of Positive Results (%)		Sum (%)	Num. of tests	Num. of Positive Results (%)		Sum (%)	
		PCR+	Rapid test+			PCR+	Rapid test+		
Total	137,704	32,253 (23.42)	17,397 (12.63)	49,650 36.05	173,037	17,577 (10.16)	20,010 (11.56)	37,587 (21.72)	-14.28

*PCR* Polymerase chain reaction, *%* percentage, *Num*. Number

**Fig. 1:**
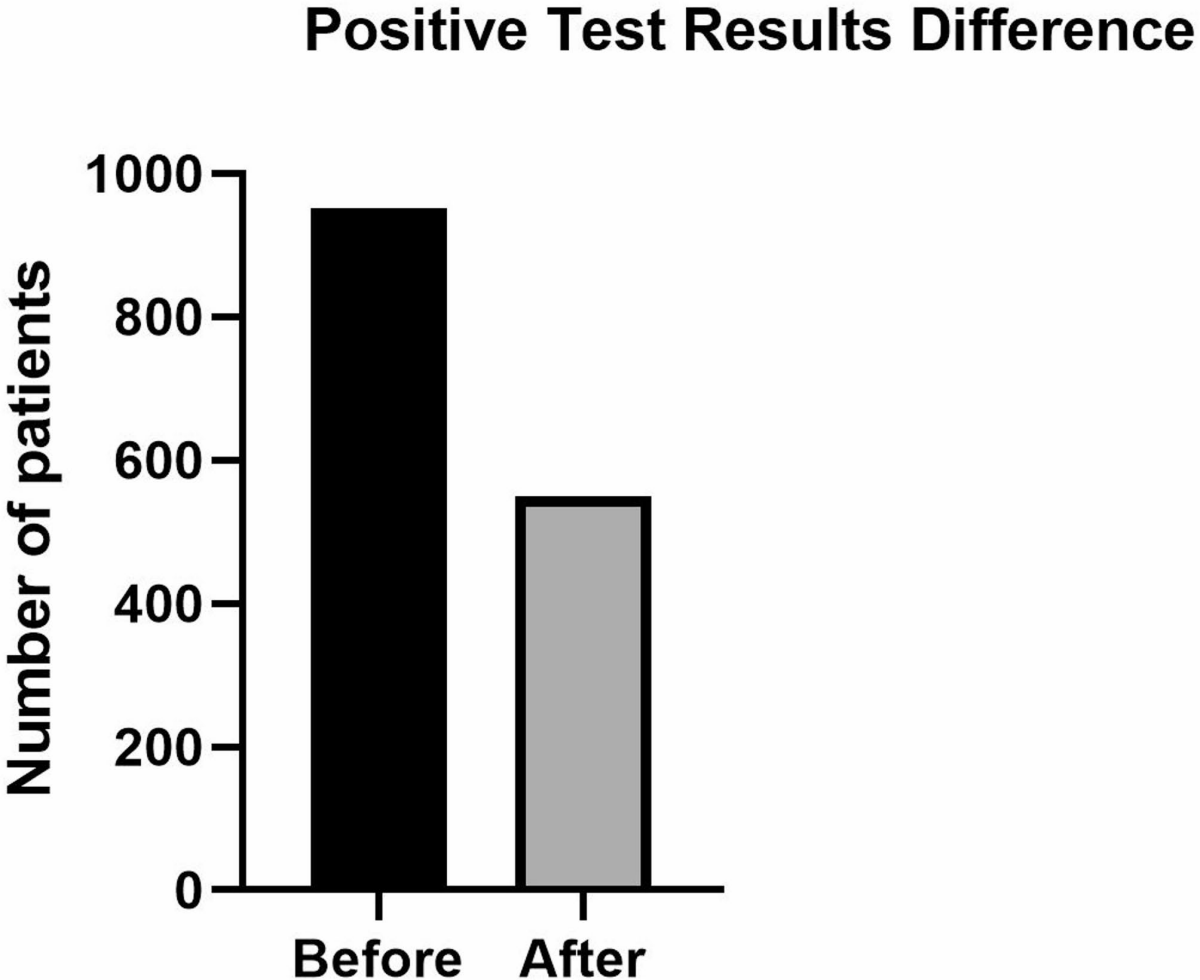
Comparison of positive test results before and after the intervention

When calculating the number of positive death cases per 1,000 people, the study recorded a total of 2,528 positive deaths, corresponding to 0.9 per 1,000 population (or 0.09% of the total population). The findings from the present study indicated significant improvements post-intervention, with the average difference in positive laboratory cases reducing from 951.4 to 550 patients per day, resulting in a mean difference of − 400.69 (Fig. 1).

**Fig. 2:**
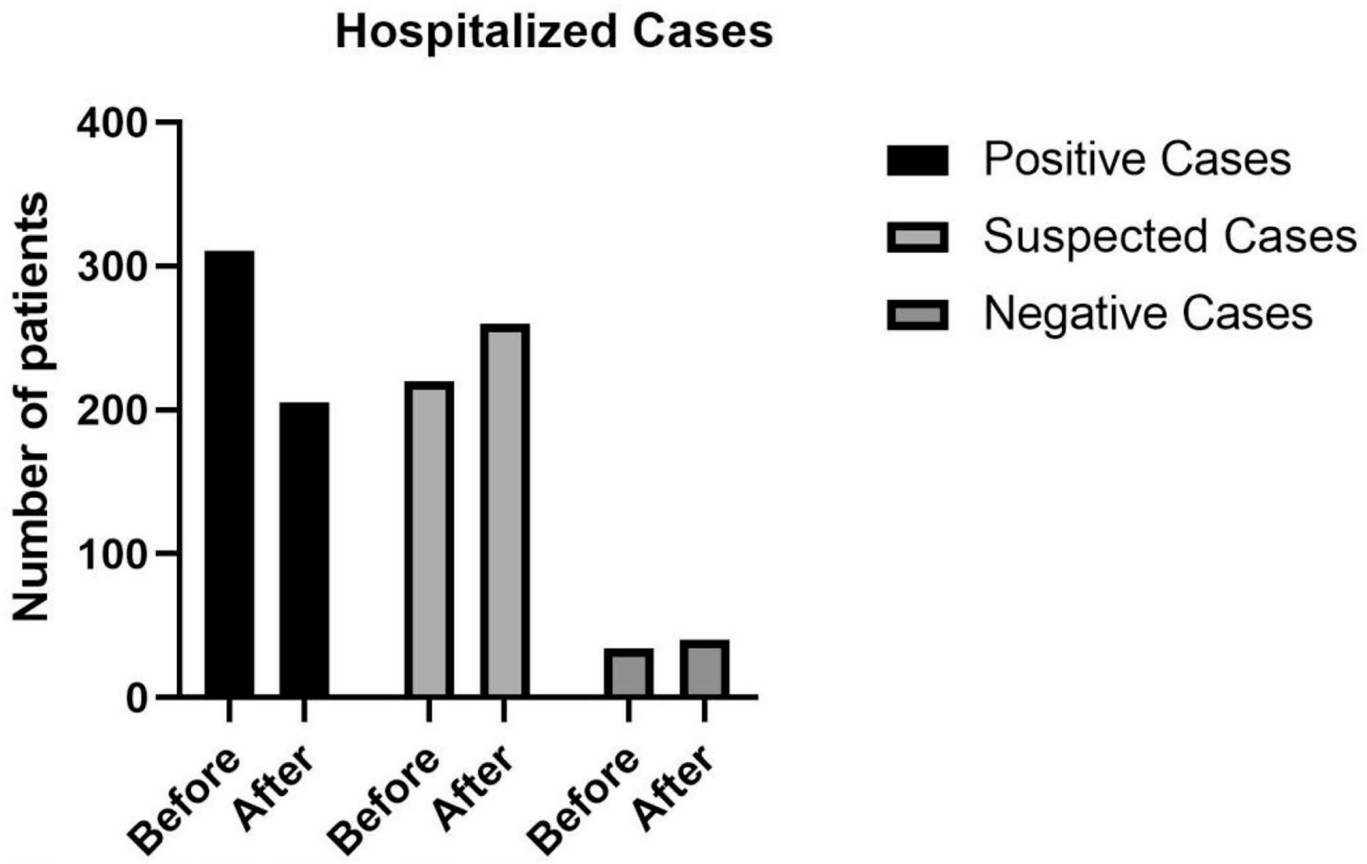
Comparison of positive, suspected, and negative cases before and after the intervention

Average daily hospitalizations decreased and positive cases recorded daily reduced from 310.48 to 205.26, suspected cases however increased after the intervention from 219.89 to 259.98 and daily negative cases increased from 34.37 to 40.12 (Fig. 2).

**Fig. 3:**
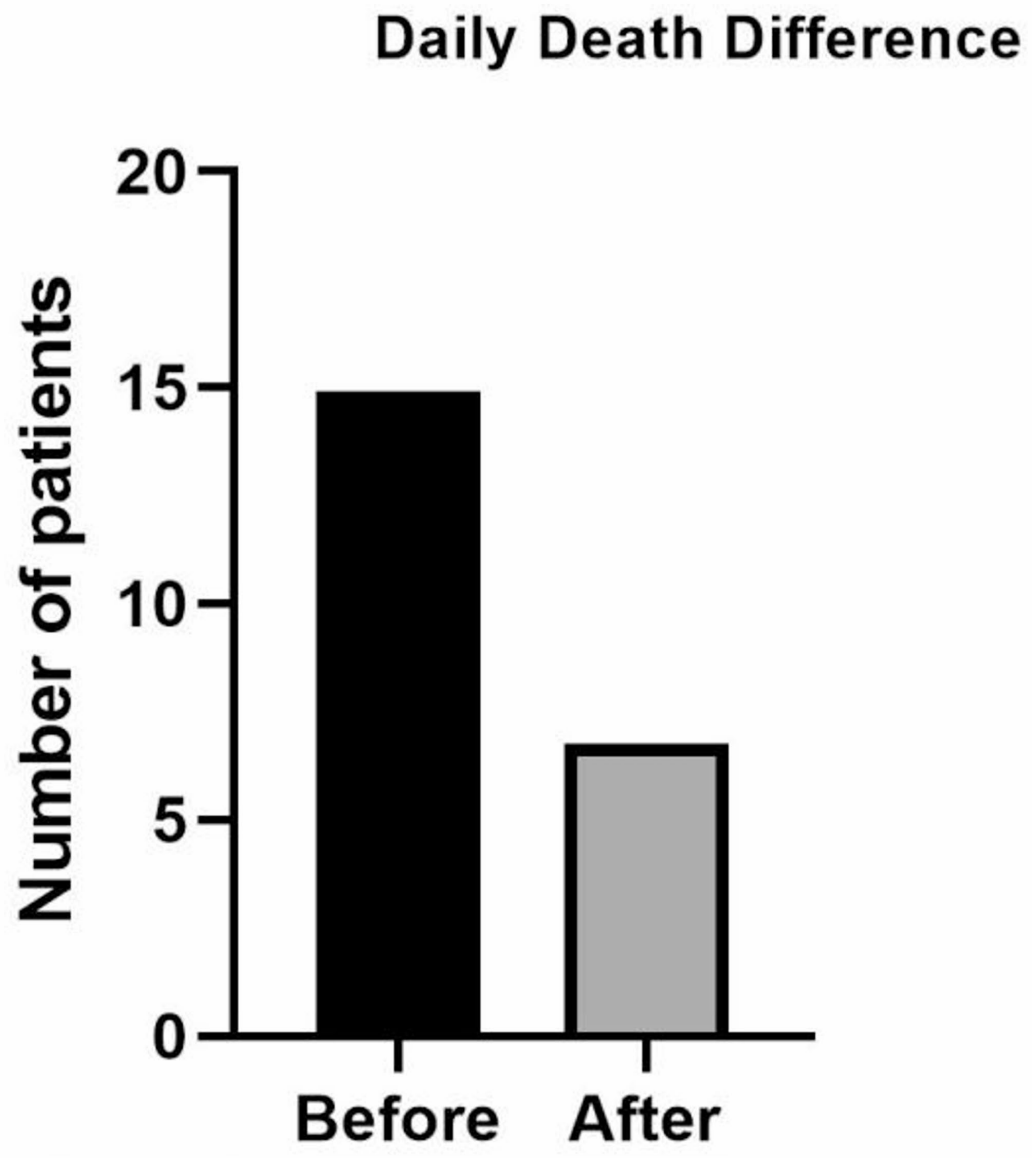
Comparison of daily death differences before and after the intervention

Figure 3 visually represents the daily average difference in COVID-19 deaths before and after the intervention, underscoring the positive impact of the measures implemented which reduced daily recorded death to approximately 8.

**Fig. 4:**
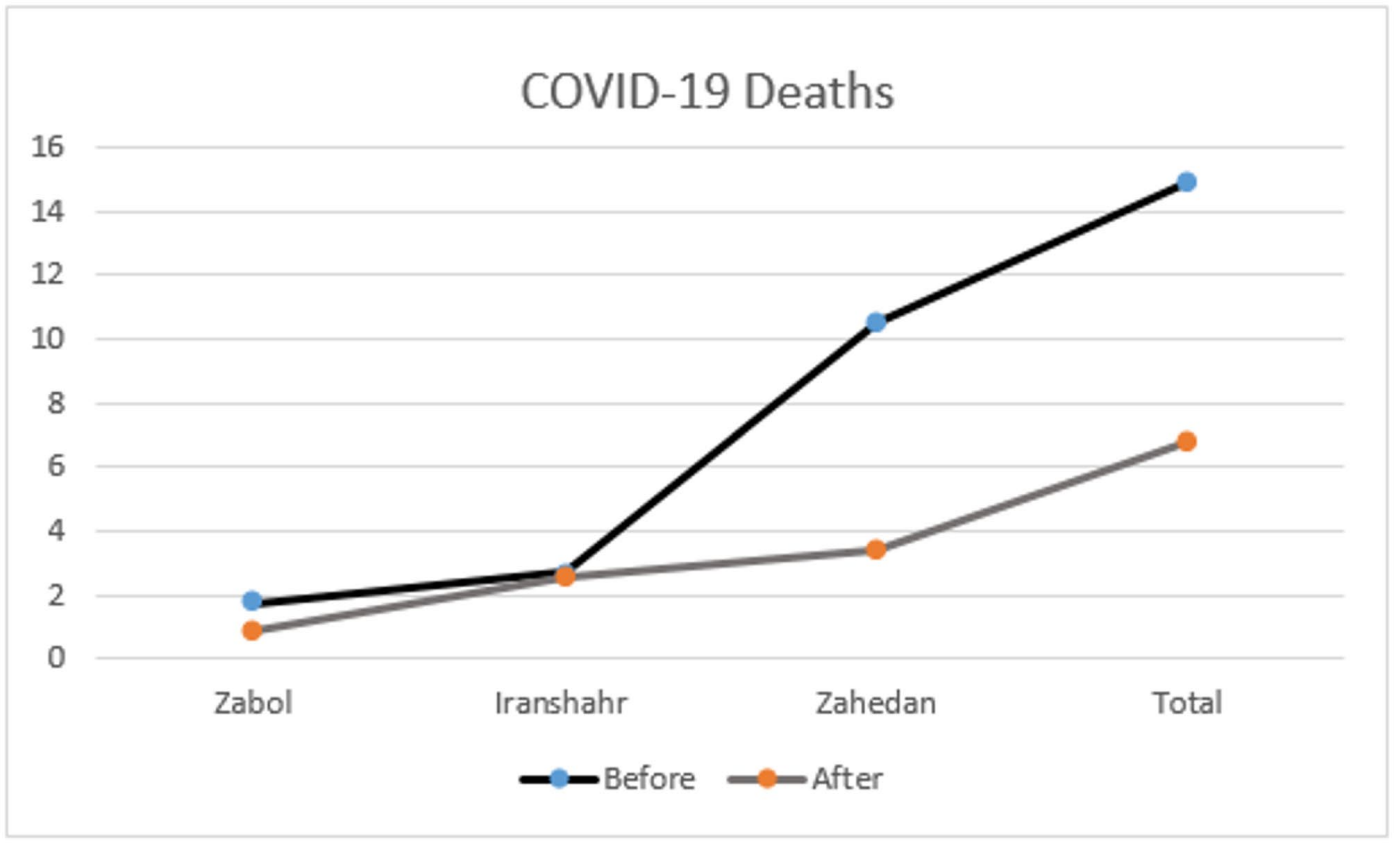
Trend comparison of daily differences in positive death cases before and after the intervention across various areas and in total

A graphical representation of the data obtained from the provinces where the intervention took place showed that in Zabol, there was a reduction COVID-19 related deaths from 2 to 1 incident per day, In Iranshahr, there was no remarkable decrease in the number of deaths prior to and after the intervention. In Zahedan however, COVID-19 related mortality decreased from 10.5 to 3.5 deaths recorded daily after the intervention process was employed (Fig. 4).

In the regression analysis, logistic regression was applied to assess the outcome of testing positive (yes/no), with predictors including the time period (pre/post-intervention), demographics, and the number of tests conducted. The results indicated a significant decrease in the odds of testing positive after the intervention, with an odds ratio (OR) of approximately 0.5, suggesting a 50% reduction in odds. Additionally, Poisson regression was utilized to evaluate daily counts of hospital admissions and deaths. The incidence rate ratio (IRR) for deaths also decreased to 0.4 post-intervention, indicating a 60% decrease in the rate of deaths.

## Discussion

This study emphasizes the importance of community-based interventions in addressing the consequences of COVID-19 with high population coverage. The significant improvements observed following targeted measures highlight the effectiveness of localized strategies in curbing infection rates and enhancing public health. This underscores the critical role of community engagement and neighborhood initiatives in managing health crises. Overall, the findings reflect a successful collaborative response to the pandemic, showcasing the potential for effective health strategies tailored to specific populations. Multiple interventions, including increasing hospital and laboratory capacities, implementing movement restrictions, promoting social distancing, hospitalizing confirmed cases, quarantining close contacts at home, and conducting public symptom assessments (door-to-door) over a quarterly period at the beginning of the epidemic, also contributed to reducing the incidence of confirmed cases and controlling the COVID-19 outbreak [11].

A review of the effectiveness of other interventions implemented in the country shows that, despite the unintended economic effects and the sharp increase in new cases of infection following the Nowruz Holiday lockdown (March 20, 2020), including travel restrictions and the closure of welfare and recreation centers, the implementation of soft interventions such as face masking and social distancing during subsequent waves of the epidemic significantly reduced new cases and deaths from COVID-19 [10, 12]. The results of a systematic review show that the effectiveness of social distancing depends on timely implementation and community acceptance [13]. Studies show that, despite the effectiveness of hand hygiene interventions, face masking, and social distancing measures such as school closures in controlling epidemics, the impact of each of these interventions alone is negligible in low- and middle-income countries. Their effectiveness depends on determinants such as knowledge and attitudes, behavior change, self-efficacy, and timing of implementation. Therefore, multiple community-based interventions are recommended in these regions [14, 15].

Adherence to mitigation measures is influenced by social and ethical norms, and education on public health risks and how to prevent COVID-19 increases adherence. Also, understanding the social norms in different communities in cases of quarantine and isolation is a key to the proper implementation of interventions [16]. Mass testing, hospitalization, and isolation of positive cases at home or hospital, based on clinical and cultural conditions was one of the most important interventions in this study. The results showed that the daily average of positive laboratory cases after the intervention decreased by about 15%. While cutting the transmission chain and controlling the clusters requires mass testing and contact tracking, the low number of diagnostic tests performed in the country has been reported as a major challenge [17].

The establishment of integrated vaccination centers in partnership with various organizations, along with the achievement of relatively high vaccination rates across different age groups in a short time frame, were key measures in reducing COVID-19-related hospitalizations and deaths in this study. Other studies also highlight the effectiveness of vaccination in preventing the disease among various age demographics, as well as in reducing hospitalizations and ICU admissions for vaccinated individuals [18, 19]. Continuation of non-pharmacological interventions for at least one year after the start of vaccination is recommended to prevent local outbreaks [20]. Community-based interventions play an important role in reducing the transmission and control of disease outbreaks. Community-based interventions are not different solutions, but they are complementary, and better results will be achieved if they are integrated and implemented in different communities. Also, supporting these interventions with appropriate socioeconomic considerations and cultural factors, instead of adopting a patriarchal approach, leads to greater social acceptance [21, 22].

### Strengths and limitations

The study’s strengths lie in its comprehensive and collaborative approach to managing a public health crisis, supported by robust data collection and analysis. The involvement of multiple sectors—health-care, education, and community organizations—facilitated a more coordinated response, maximizing resource utilization and community outreach. These factors contributed to significant improvements in health metrics, showcasing the effectiveness of the intervention during the COVID-19 outbreak in Iran.

The study has some potential weaknesses, including its quasi-experimental design without a control group, which limits causal inferences. The short three-week intervention period may not capture long-term effects, while reliance on descriptive statistics can oversimplify data analysis and introduce biases in reporting. Cultural barriers and socioeconomic disparities could confound the results, and variability in implementation might affect outcomes. Additionally, the limited outcome metrics assessed do not provide a comprehensive view of public health impacts. The findings may lack generalization to other populations, and the absence of long-term follow-up data hinders the evaluation of sustained intervention effects.

This study suggests that further research should evaluate the effectiveness of continued non-pharmaceutical interventions in conjunction with vaccination across different target and age groups.

A study indicated that the mean score of family caregiver burden was 2.61 ± 0.6, which places the severity of this burden in a moderate range. Statistical differences in the mean scores of caregiver burden were observed based on gender, duration of treatment, age, and employment status. Being female, married, employed, elderly, having low income, and poor education—significantly influenced the family caregiver burde. By increasing health managers’ awareness of the extent of this burden, and developing economic, social, and psychological support programs, the burden of illness among caregivers during the COVID-19 outbreak can be reduced [23].

## Conclusion

The study highlights the importance of community-based interventions in managing COVID-19 by reducing infection rates and improving public health through targeted local strategies. Despite challenges such as economic impacts and public compliance, interventions like masking have proven effective. Timely implementation, community acceptance, and high vaccination rates are crucial, along with ongoing adherence to non-pharmaceutical measures for sustained virus control. The research recommends a comprehensive approach to public health crises, emphasizing the need for tailored community interventions alongside vaccination campaigns for optimal outcomes. Further exploration of their long-term efficacy across diverse populations is warranted.

The researchers suggest a strategy to improve conditions for beneficiaries through these tailored community interventions, which should be localized and culturally sensitive, focusing on socioeconomic factors. Maintaining high vaccination rates while adhering to safety measures is essential. Collaboration with community leaders is crucial for promoting education and compliance, thereby preventing outbreaks and ensuring optimal health outcomes in various communities. Future research on COVID-19 caregiver burden should focus on longitudinal studies, larger, diverse samples, and comparative studies with other chronic illnesses. Qualitative methods like interviews can provide deeper insights into caregiver experiences. Evaluating support programs and technology, cultural influences on caregiving, and long-term mental health outcomes are crucial areas for investigation. Investigating healthcare providers’ roles and factors contributing to caregiver resilience can enhance coping strategies for these individuals.

## Data Availability

No datasets were generated or analysed during the current study.
